# Kinematic Viscosity of Multicomponent FeCuNbSiB-Based Melts

**DOI:** 10.3390/nano11041042

**Published:** 2021-04-19

**Authors:** Yuri N. Starodubtsev, Vladimir S. Tsepelev, Nadezhda P. Tsepeleva

**Affiliations:** 1 Research Center for Physics of Metal Liquids, Ural Federal University, Mira Str. 19, 620002 Ekaterinburg, Russia; yunstar@mail.ru (Y.N.S.); n.p.konovalova@urfu.ru (N.P.T.); 2 Research and Production Enterprise, Tatishchev Str. 92, 620131 Ekaterinburg, Russia

**Keywords:** Arrhenius equation, activation energy, kinematic viscosity, liquid multicomponent alloy, liquid metal, liquid–liquid structure transition

## Abstract

The work investigated the temperature dependences of the kinematic viscosity for multicomponent melts of nanocrystalline soft magnetic alloys. It is shown that there is a linear relationship between the reduced activation energy of viscous flow *E_a_*·(*RT*)^−1^ and the pre-exponential factor ν_0_. This ratio is universal for all quantities, the temperature dependence of which is expressed by the Arrhenius equation. It is shown that the activation energy of a viscous flow is linearly related to the cluster size on a natural logarithmic scale, and the melt viscosity increases with decreasing cluster size. The change in the Arrhenius plot in the anomalous zone on the temperature dependence of viscosity can be interpreted as a liquid–liquid structure transition, which begins with the disintegration of clusters and ends with the formation of a new cluster structure.

## 1. Introduction

Nanocrystalline soft magnetic materials are used for the manufacture of magnetic systems for various electrical devices [1]. The first and most widely used material was the classical nanocrystalline Finemet alloy with the chemical composition Fe_73.5_Cu_1_Nb_3_Si_13.5_B_9_ [2]. To obtain special magnetic properties, the classical composition can be modified with various chemical elements Ni, Co, Mo, V, Cr and others.

Nanocrystalline soft magnetic materials are multicomponent alloys. When melting a multicomponent alloy, a mushy zone is formed between the solidus and liquidus temperatures, in which the liquid and solid phases coexist. After transition to a liquid state, the melt inherits the short-range order, which is associated with a more stable solid phase. The structural component of the melt is clusters, the size of which depends on temperature [3]. Heating of melt above the critical temperature *T_k_* followed by quenching makes it possible to obtain an amorphous state with a more homogeneous structure. Such an amorphous material has a larger molar volume [4], enthalpy of crystallization [4,5] and plasticity [6]. This proves that the heredity of the melt structure is preserved in the amorphous solid state.

The critical temperature is revealed on the temperature dependence of the melt viscosity, which is very sensitive to structural changes [7,8,9]. The Arrhenius equation determines the rate of the process, in particular, the rate of diffusion [10]. Viscosity is related to the mobility of particles participating in a viscous flow, therefore it is inversely proportional to the diffusion coefficient [11], and:

(1) $$\nu =\nu_{0}e^{\frac{E_{a}}{RT}},$$

where ν is the kinematic viscosity (m^2^·s^−1^), ν_0_ is a pre-exponential factor with the dimension of the kinematic viscosity, *E_a_* is the activation energy of the viscous flow (J·mol^−1^), *R* is the gas constant (J·K^−1^·mol^−1^), *T* is the absolute temperature (K). At constant of ν_0_ and *E_a_*, the melt viscosity decreases with increasing temperature. After taking the logarithm we obtain Arrhenius plot:

(2) $$\ln\nu =\ln\nu_{0}+\frac{E_{a}}{RT}.$$

Thus, the logarithm of the kinematic viscosity is a linear function of the inverse absolute temperature. Full differential can be found from Arrhenius plot:

(3) $$d\ln\nu =-\frac{E_{a}dT}{RT^{2}},$$

and the activation energy *E_a_*

(4) $$E_{a}=R\frac{d\ln\nu }{dT^{-1}}.$$

Above the critical temperature, the Arrhenius plot becomes nonlinear, and the activation energy changes [12,13]. The change in the activation energy is associated with the liquid–liquid structure transition (LLST) [14], which is reflected in the thermophysical properties of the melt [15,16].

The article investigates the Arrhenius equation for the kinematic viscosity of simple liquid metals at melting point and multicomponent FeCuNbSiB-based melts. The main aim was to determine the relationship between the activation energy of a viscous flow with the cluster size and to interpret the anomalous Arrhenius plot for multicomponent melts.

## 2. Materials and Methods

The experimental thermophysical properties of simple liquid metals at a melting point, marked with the sub-index *m*, were taken from [17]. These quantities are the melting point *T_m_*, density ρ*_m_* (kg·m^−3^), molar volume *V_m_* (m^3^·mol^−1^), dynamic viscosity η*_m_* (Pa·s), activation energy *E_a_*, and pre-exponential factor η_0*m*_ (Pa·s). The atomic size *a* (m) was taken as:

(5) $$a=\sqrt[{3}]{\frac{V_{m}}{N_{A}}},$$

where *N_A_* is Avogadro constant (mol^−1^). The kinematic viscosity at the melting point and the pre-exponential factor were calculated from the relations η*_m_* = ν*_m_*ρ*_m_* и η_0*m*_ = ν_0*m*_ρ*_m_*. The approximation to the linear dependence was estimated using regression analysis, and the degree of approximation was compared using the adjusted coefficient of determination *R*^2^*_adj_*. Metals were divided into four groups: alkali and alkaline earth, rare earth, and transition metals, as well as metals of 12–14 groups of the periodic table.

Activation energy and pre-exponential factor for multicomponent melts were taken from our previous works [1,18,19,20,21]. They were FeCuNbSiB-based melts, namely Fe_72.5_Cu_1_Nb_2_Mo_1.5_Si_14_B_9_ and Fe_73.5_Cu_1_M_3_Si_13.5_B_9_ where M = Nb, Mo, V. For binary Fe-Si alloys, we used data from [22].

Especially for this work, the temperature dependences of the kinematic viscosity in Fe_84.5_Cu_0.6_Nb_0.5_Si_1.5_B_8.6_P_4_C_0.3_ and Fe_72.5-*x*_Ni*_x_*Cu_1_Nb_2_Mo_1.5_Si_14_B_9_ melts with Ni content 2.5, 6.3 and 12.7 at% were investigated. The alloys were melted in a vacuum induction furnace at a temperature of 1820 K and cooled in a flat mold [23]. The kinematic viscosity was measured by the method of torsional vibrations in an atmosphere of pure helium at a pressure of 10^5^ Pa [24]. During heating and cooling, the temperature of the melt was changed with a step of 30 K. Before measurement, the melt was kept at a given temperature for 8 min to stabilize the structural state. The error in measuring the kinematic viscosity was 3%. The activation energy *E_a_* and the pre-exponential factor ν_0_ were calculated from the temperature dependence of the viscosity in the linear sections of the Arrhenius plot. The results for the Fe_72.5-*x*_Ni*_x_*Cu_1_Nb_2_Mo_1.5_Si_14_B_9_ melts are presented in Table 1. 

## 3. Consequences of Arrhenius Equation

From Equation (2) it follows that at a fixed temperature, the activation energy *E_a_* and the pre-exponential factor ν_0_ are related to each other by the equation:

(6) $$\frac{E_{a}}{RT}=C-\ln\nu_{0},$$

where *E_a_*·(*RT*)^–1^ is the activation energy reduced to a unit of thermal energy *RT*, *C* is a constant that generally depends on temperature.

In the hole model it is assumed that the liquid has a lattice structure, at least in the first coordination sphere [25], and every atom vibrates inside a limited space. The space available for the movement of an atom or particle is the free volume v*_f_* (m^3^). The motion of one layer of liquid relative to another can occur due to the transition of a particle (atom) of size *a* from the equilibrium state to a free site (hole). In the theory of the transition state, such a mechanism creates a dynamic viscosity η (Pa·s) [26]:

(7) $$\eta =\frac{N_{A}}{V_{m}}{\left(2\pi mk_{B}T\right)}^{1/2}v_{f}^{1/3}e^{\frac{E_{a}}{RT}},$$

where *m* is the mass of an atom or particle (kg). Taking into account η = νρ, where ρ is the density, we obtain the kinematic viscosity in the form [20]:

(8) $$\nu ={\left(\frac{2\pi k_{B}T}{a\rho }\right)}^{1/2}{\left(\frac{v_{f}}{v}\right)}^{1/3}e^{\frac{E_{a}}{RT}},$$

where v*_f_*/v—the relative free volume, v is the atomic volume (m^3^), and *V_m_* = v*N_A_*.

After substituting the pre-exponential factor from (8) into (6), we obtain the relationship between the reduced activation energy and the particle size participating in a viscous flow:

(9) $$\frac{E_{a}}{RT}=C_{1}+0.5\ln a,$$

where *C*_1_ is a constant, which in general also depends on temperature. Thus, the activation energy of viscous flow is linearly related to the particle size on a natural logarithmic scale.

## 4. Simple Liquid Metals

**Figure 1:**
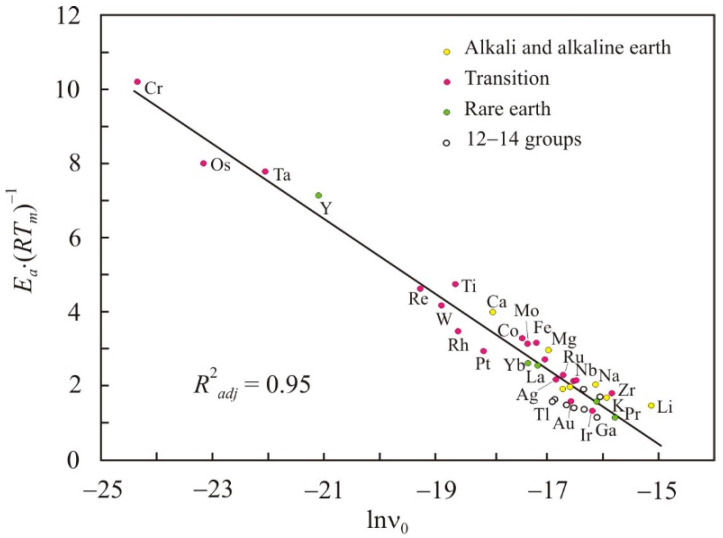
Relation of the reduced activation energy *E_a_*·(*RT_m_*)^−1^ with the pre-exponential factor in the natural logarithmic scale lnν_0_ for liquid metals at the melting point *T_m_*.

Figure 1 shows the dependence of the reduced activation energy *E_a_*·(*RT_m_*)^−1^ on the pre-exponential factor in the natural logarithmic scale lnν_0_ for simple liquid metals at the melting point. In accordance with (6), there is a linear relationship with the adjusted coefficient of determination *R*^2^*_adj_* = 0.95 and the constant *C* = −14.7.

The relative free volume in Equation (8) can be estimated according to Lindemann [27]. At the melting point, the average distance between the centers of vibrating atoms increases by 0.1*a*, and the relative free volume is 0.158. This allows us to represent the kinematic viscosity at the melting point in the form:

(10) $$\nu_{m}=1.35{\left(\frac{k_{B}T_{m}}{a\rho_{m}}\right)}^{1/2}e^{\frac{E_{a}}{RT_{m}}},$$

Comparison of (1) and (10) implies that the quantity

(11) $$\nu_{0m}^{cal}=1.35{\left(\frac{k_{B}T_{m}}{a\rho_{m}}\right)}^{1/2}$$

is the pre-exponential factor at the melting point of the simple liquid metals. Comparison of the calculated pre-exponential factor ν_0*m*_*^cal^* with the experimental ν_0*m*_*^ex^* for liquid metals at the melting point shows that there are metals for which this ratio is much greater than 1.

Formula (11) includes the melting point *T_m_*, the density ρ*_m_*, and the atomic size *a*. The melting point and density are measured by experimental methods with great accuracy. The discrepancy between the experimental and calculated pre-exponential factors can be resolved if we assume that clusters rather than atoms participate in the viscous flow. Indeed, it was shown in [28,29,30] that simple liquid metals can have a cluster structure. Due to coalescence, clusters can form even larger associates—fractal clusters [31,32].

We find the assumed cluster size *a_c_* from the relation:

(12) $$a_{c}={\left(\frac{1.35}{\nu_{0m}^{ex}}\right)}^{2}\frac{k_{B}T_{m}}{\rho_{m}}$$

**Figure 2:**
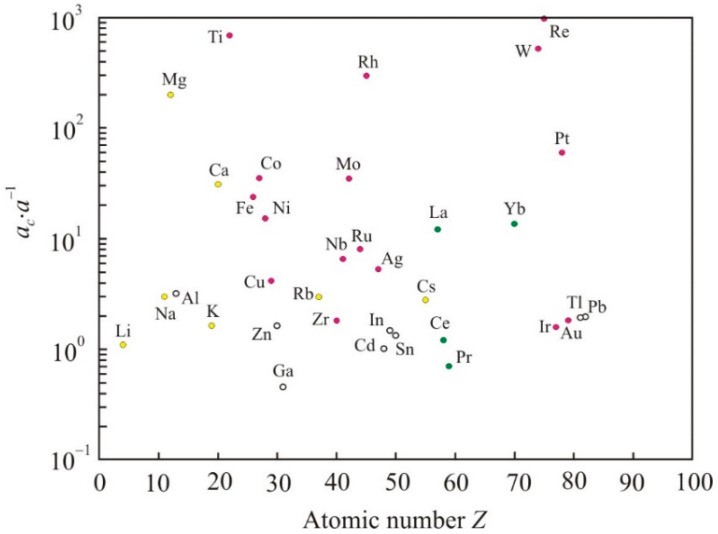
Ratio of the calculated cluster size to the atomic size *a_c_*/*a* for liquid metals at the melting point.

Figure 2 shows the ratio of the calculated cluster size *a_c_* to the atomic size *a*. For Cr, Ta, Os, and Y, the *a_c_*/*a* is more than 1000 and in Figure 2 is not shown. Metals Mg, Ti, W, Re, Rh have a ratio of more than 100. All of these metals have a high reduced activation energy *E_a_*·(*RT_m_*)^−1^. Many of them have high cohesive energy, but there is no direct relationship between the calculated cluster size and the cohesive energy. Thus, if we proceed from the transition state theory, then the viscous flow of many liquid metals at melting point can be associated with the motion of clusters.

If we exclude from consideration metals with a large *a_c_*/*a* ratio of more than about 5, then for the remaining metals the reduced activation energy *E_a_*·(*RT_m_*)^−1^ changes slightly near the average value of 1.65. For these elements, instead of (10), we can write:

(13) $$\nu_{m}\propto {\left(\frac{k_{B}T_{m}}{a\rho_{m}}\right)}^{1/2}$$

**Figure 3:**
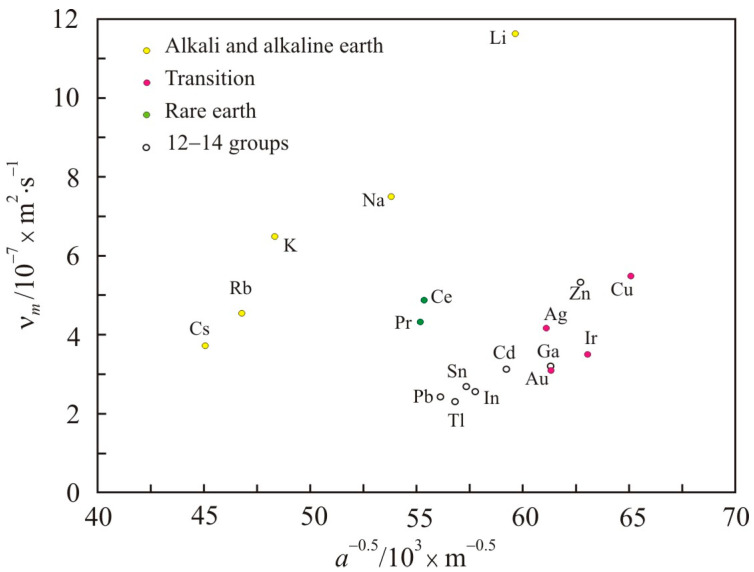
Dependence of the kinematic viscosity at the melting point ν*_m_* on the quantity of *a*^−0.5^, where *a* is the atomic size.

Figure 3 shows the dependence of the kinematic viscosity at the melting point ν*_m_* on atomic size *a* for the selected group of elements. It is seen that the viscosity increases with increasing *a*^−0.5^, i.e., with decreasing atomic size. In a finely dispersed medium, the interaction energy between particles and viscosity are higher [33]. The nanofluid viscosity also increases with decreasing particle size at a fixed particle concentration [34].

## 5. Multicomponent Melts

**Figure 4:**
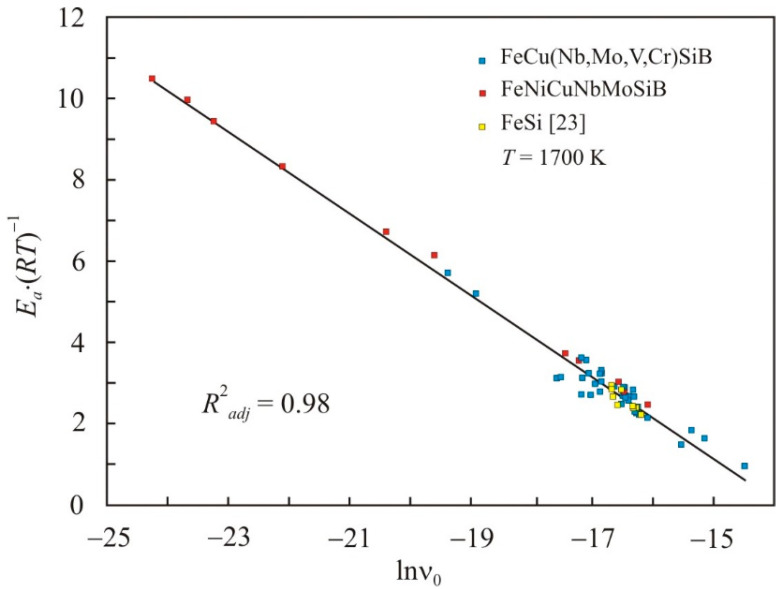
Relation of the reduced activation energy *E_a_*·(*RT*)^−1^ with the pre-exponential factor in the natural logarithmic scale lnν_0_ for multicomponent FeCuNbSiB-based and binary Fe-Si melts at a temperature of 1700 K.

Figure 4 shows the dependence of the reduced activation energy on the pre-exponential factor in a natural logarithmic scale for multicomponent FeCuNbSiB-based and binary Fe-Si melts [23] at a temperature of *T* = 1700 K. The adjusted coefficient of determination *R*^2^*_adj_* for the linear relationship (6) has a high value of 0.98, and the constant *C* = −13.9. Figure 4 shows that the linear dependence repeats the same dependence for simple liquid metals in Figure 1, but with another constant *C*. These results confirm that there is a relationship between the reduced activation energy and the pre-exponential factor, which can be represented in the form of relation (6). This relationship also takes place for all physical quantities, the temperature dependence of which can be represented in the form of the Arrhenius equation.

**Figure 5:**
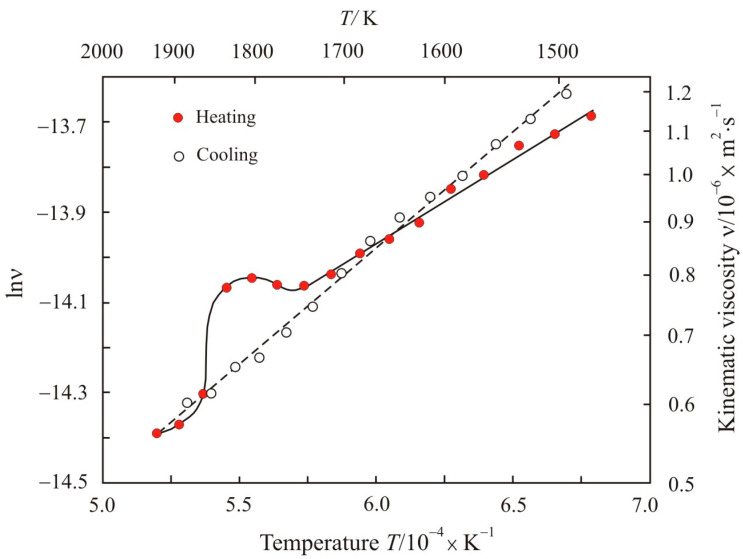
Kinematic viscosity in natural logarithmic scale lnν as a function of the inverse absolute temperature 10^4^ × *T*^−1^ upon heating to the maximum temperature 1920 K and cooling the Fe_84.5_Cu_0.6_Nb_0.5_Si_1.5_B_8.6_P_4_C_0.3_ melt.

Figure 5 shows the kinematic viscosity on a natural logarithmic scale lnν as a function of the inverse absolute temperature 10^4^ × *T*^−1^ upon heating to *T* = 1920 K and cooling the Fe_84.5_Cu_0.6_Nb_0.5_Si_1.5_B_8.6_P_4_C_0.3_ melt. It can be seen that upon heating to the critical temperature *T_k_* = 1740 K, the dependence is a straight line and it corresponds to the Arrhenius plot at constant activation energy and pre-exponential factor. With an increase in temperature, the viscosity first increases, and then decreases and passes to a trajectory close to the low-temperature region. The anomalous zone is located in the temperature range 1740–1870 K.

Before the measurement, the melt was kept at a predetermined temperature for 8 min to stabilize the structural state. Therefore, the state of the melt will be considered close to equilibrium. This is also evidenced by a smooth change in viscosity with temperature. A change in the slope of the Arrhenius plot in the anomalous zone indicates a decrease in the activation energy. In accordance with relation (9), a decrease in the activation energy is associated with a decrease in the cluster size up to decomposition into individual atoms. With a further increase in temperature in the anomalous zone, the slope of the Arrhenius plot and the activation energy increase. This stage can be associated with the formation of new clusters, which have a different structure and, possibly, a different chemical composition. The newly formed cluster structure is close to the state, which is reached after holding the melt at the maximum temperature. Thus, the change in the Arrhenius plot in the anomalous zone and the ratio between the activation energy and the cluster size suggests that the anomaly in the temperature dependence of the multicomponent melt is associated with LLST.

A similar anomaly in the temperature dependence of viscosity was observed in other melts [9,35,36]. The appearance of an anomaly in boron-containing melts was associated with the rearrangement of FeB- and Fe_2_B-based clusters, which transform with increasing temperature into Fe_4_B- [37] or Fe_3_B-based clusters [38].

During cooling, the dependence of lnν on 10^4^ × *T*^−1^ is linear over the entire temperature range, see Figure 5. The linear Arrhenius plot shows that the viscous flow does not change qualitatively. In addition, upon cooling, the slope of the Arrhenius plot is greater than upon heating, and the corresponding activation energies are 31.3 and 39. kJ·mol^−1^. It follows from this that the clusters that formed at a maximum temperature of 1920 K are larger.

**Figure 6:**
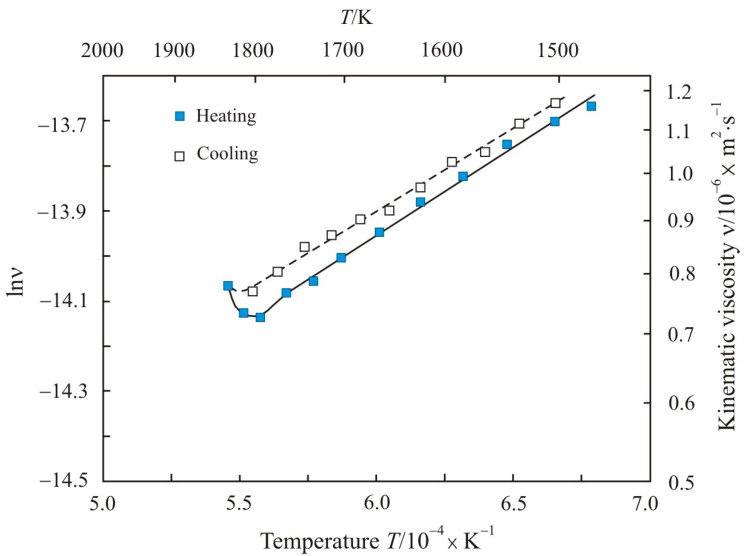
Kinematic viscosity in natural logarithmic scale lnν as a function of the inverse absolute temperature 10^4^ × *T*^−1^ upon heating to the maximum temperature 1820 K and cooling the Fe_84.5_Cu_0.6_Nb_0.5_Si_1.5_B_8.6_P_4_C_0.3_ melt.

Figure 6 shows the kinematic viscosity on a natural logarithmic scale lnν as a function of the inverse absolute temperature 10^4^ × *T*^−1^ during heating to a maximum temperature 1820 K and cooling Fe_84.5_Cu_0.6_Nb_0.5_Si_1.5_B_8.6_P_4_C_0.3_ melt. The temperature of 1820 K falls in the middle of the anomalous zone, i.e., at the stage of decomposition of the initial cluster structure. On cooling, this melt has the lowest activation energy of 30.3 kJ·mol^−1^, which corresponds to a smaller size of clusters formed at a temperature of 1820 K at the decomposition stage.

## 6. Conclusions

In this work, the Arrhenius equation for kinematic viscosity was investigated. For the analysis, we used the experimental temperature dependences of the kinematic viscosity for multicomponent melts of nanocrystalline soft magnetic alloys, as well as the thermophysical properties of simple liquid metals at the melting point. The experimental data were compared with the viscosity obtained in the transition state theory. The aim of this work was to determine the relationship between the activation energy of a viscous melt flow and the cluster size and to interpret the anomalous Arrhenius plot for multicomponent melts. The results can be summarized as the following main conclusions.

1. There is a relationship between the reduced activation energy of viscous flow *E_a_*·(*RT*)^−1^ and the pre-exponential factor ν_0_, which can be expressed by the relation:
  $$\frac{E_{a}}{RT}=C-\ln\nu_{0}$$
  where C is a constant that generally depends on temperature. This relationship is universal for all quantities, the temperature dependence of which is expressed by the Arrhenius equation.
2. The activation energy of viscous flow is linearly related to the cluster size on a natural logarithmic scale.
3. Melt viscosity increases with decreasing cluster size.
4. The change in the Arrhenius plot in the anomalous zone can be interpreted as a liquid–liquid structure transition, which begins with the disintegration of clusters and ends with the formation of a new cluster structure.

## Figures and Tables

**Table 1:** Calculated activation energy of viscous flow *E_a_* and pre-exponential factor ν_0_ in Fe_72.5-*x*_Ni*_x_*Cu_1_Nb_2_Mo_1.5_Si_14_B_9_ melt for linear sections of Arrhenius plots.

Ni Content (at%)	Heating				Cooling			
	*T* > 1700 K		*T* < 1700 K		*T* > 1700 K		*T* < 1700 K	
	*E_a_* kJ·mol^−1^	ν_0_ × 10^−8^ m^2^·s^−1^	*E_a_* kJ·mol^−1^	ν_0_ × 10^−8^ m^2^·s^−1^	*E_a_* kJ·mol^−1^	ν_0_ × 10^−8^ m^2^·s^−1^	*E_a_* kJ·mol^−1^	ν_0_ × 10^−8^ m^2^·s^−1^
2.5	141	0.0052	35	10.3	86.8	0.307	42.6	6.38
6.3	134	0.0081	52.6	2.66	95	0.139	39.1	7.04
12.7	148	0.0029	–	–	118	0.025	50	3.31

## Data Availability

The data presented in this article is available upon request from the corresponding author.

## References

[1] Yoshizawa Y., Oguma S., Yamauchi K. (1988). New Fe-based soft magnetic alloys composed of ultrafine grain structure. J. Appl. Phys..

[2] Tsepelev V.S., Starodubtsev Y.N. (2021). Nanocrystalline soft magnetic iron-based materials from liquid state to ready product. Nanomatarials.

[3] Calvo-Dahlborg M., Popel P.S., Kramer M.J., Besser M., Morris J.R., Dahlborg U. (2013). Superheat-dependent microstructure of molten Al-Si alloys of different compositions studied by small angle neutron scattering. J. Alloys Compd..

[4] Manov V.P., Popel S.I., Buler P.I., Manukhin A.B., Komlev D.G. (1991). The influence of quenching temperature on the structure and properties of amorphous alloys. Mater. Sci. Eng. A.

[5] Tsepelev V., Starodubtsev Y., Konashkov V. (2017). Melt viscosity of the soft magnetic nanocrystalline Fe_72.5_Cu_1_Nb_2_Mo_1.5_Si_14_B_9_ alloy. EPJ Web Conf..

[6] Starodubtsev Y.N., Son L.D., Tsepelev V.S., Tyagunov G.V., Tishkin A.P., Korobka O.B. (1992). Influence of the melt heating temperature on the mechanical and magnetic properties of an amorphous ribbon. Rasplavy.

[7] Bel’tyukov A.L., Lad’yanov V.I., Shishmarin A.I., Menshikov S.G. (2014). Viscosity of liquid amorphizing alloys of iron with boron and silicon. J. Non Cryst. Solids.

[8] Dong B.S., Zhou S.X., Wang Y.G., Li Y., Qin J.Y., Li G.Z. (2018). Revealing a structure transition in typical Fe-based glass-forming alloy. J. Non Cryst. Solids.

[9] Zhao X., Wang C., Zheng H., Tian Z., Hu L. (2018). The role of liquid-liquid transition in glass formation of CuZr alloys. Phys. Chem. Chem. Phys..

[10] Stiller W. (2000). Arrhenius Equation and Non-Equlibrium Kinetics.

[11] Frenkel J. (1975). Kinetic Theory of Liquids.

[12] Ward A.G. (1937). The viscosity of pure liquids. Trans. Faraday Soc..

[13] Chikova O.A., Tkachuk G.A., V’yukhin V.V. (2019). Viscosity of Cu-Ni melts. Rus. J. Phys. Chem. A.

[14] Tanaka H. (2000). General view of a liquid-liquid phase transition. Phys. Rev. E.

[15] Vasin M.G., Lad’yanov V.I. (2003). Structural transitions and nonmonotonic relaxation processes in liquid metals. Phys. Rev. E.

[16] He Y., Li J., Wang J., Kou H., Beagunon E. (2017). Liquid-liquid structure transition and nucleation in undercooled Co-B eutectic alloys. Appl. Phys. A.

[17] Iida T., Guthrie R.I.L. (2015). The Thermophysical Properties of Metallic Liquids.

[18] Konashkov V.V., Tsepelev V.S., Belozerov V.Y., Starodubtsev Y.N. (2012). Influence of smelting technology on properties of amorphizing Fe-S-B melts. Steel Transl..

[19] Tsepelev V., Starodubtsev Y., Konashkov V., Wu K., Wang R. (2019). Melt viscosity of nanocrystalline alloys in the model of free volume. J. Alloys Compounds.

[20] Tsepelev V.S., Starodubtsev Y.N., Wu K.M., Kochetkova Y.A. (2020). Nanoparticles size in Fe_73.5_Cu_1_Mo_3_Si_13.5_B_9_ melt. Key Eng. Mater..

[21] Kochetkova Y.A., Starodubtsev Y.N., Tsepelev V.S. (2020). Kinematic viscosity of melt prepared from an amorphous Fe_72.5_Cu_1_Nb_2_Mo_1.5_Si_14_B_9_ ribbon. IOP Conference Series: Materials Science and Engineering.

[22] Bel’tyukov A.L., Lad’yanov V.I., Shishmarin A.I. (2014). Viscosity of Fe-Si melts with silicon content up to 45 at%. High. Temp..

[23] Tsepelev V.S., Starodubtsev Y.N., Wu K.M. (2019). Influence of Ni on crystallization and magnetic properties of Fe_72.5-x_Ni_x_Cu_1_Nb_2_Mo_1.5_Si_14_B_9_ alloys. J. Cryst. Growth.

[24] Tsepelev V., Konashkov V., Starodubtsev Y., Belozerov Y., Gaipisherov D. (2012). Optimum regime of heat treatment of soft magnetic amorphous materials. IEEE Trans. Magn..

[25] De With G. (2013). Liquid-State Physical Chemistry. Fundamentals, Modeling, and Applications.

[26] Glasstone S., Laidler K., Eyring H. (1941). The Theory of Rate Processes. The Kinetics of Chemical Reactions, Viscosity, Diffusion and Electrochemical Phenomena.

[27] Lindemann F.A. (1910). Über die Berechnung molekularer Eigenfrequenzen. Phys. Z..

[28] Zhai Q., Luo J., Zhao P. (2004). Effect of thermal cycle on liquid structure of pure iron at just above its melting point. ISIJ Int..

[29] Louzguine-Luzgin D.V., Miyama M., Nishio K., Tsarkov A.A., Greer A.L. (2019). Vitrification and nanocrystallization of pure liquid Ni studied molecular-dynamics simulation. J. Chem. Phys..

[30] Song L., Tian X., Yang Y., Qin J., Li H., Lin X. (2020). Probing the microstructure in pure Al & Cu melts: Theory meets experiment. Front. Chem..

[31] Smirnov B.M. (1990). The properties of fractal clusters. Phys. Rep..

[32] Yang M.H., Li J.H., Liu B.X. (2018). Fractal analysis on the cluster network in metallic liquid and glass. J. Alloys Comp..

[33] Baum B.A. (1979). Metal Liquids.

[34] Koca H.D., Doganay S., Turgut A., Tavman I.H., Saidur R., Mahbubul I.M. (2018). Effect of particles size on viscosity of nanofluids: A review. Renew. Sustain. Energy Rev..

[35] Dahlborg U., Calvo-Dahlborg M., Popel P.S., Sidorov V.E. (2000). Structure and properties of som glass-forming liquid alloys. Eur. Phys. J. B.

[36] Chikova O., Sinitsin N., Vyukhin V., Chezganov D. (2019). Microheterogeneity and crystallization conditions of Fe-Mn melts. J. Cryst. Growth.

[37] Beľtyukov A.L., Goncharov O.Y., Laďyanov V.I. (2017). Features of polytherms of the viscosity of Fe-B melts. Rus. J. Phys. Chem..

[38] Dong B., Zhou S., Qin J., Li Y., Chen H., Wang Y. (2018). The hidden disintegration of cluster heterogeneity in Fe-based glass-forming. Prog. Nat. Sci. Mater..

